# Analyzing the impact of social factors on homelessness: a Fuzzy Cognitive Map approach

**DOI:** 10.1186/1472-6947-13-94

**Published:** 2013-08-23

**Authors:** Vijay K Mago, Hilary K Morden, Charles Fritz, Tiankuang Wu, Sara Namazi, Parastoo Geranmayeh, Rakhi Chattopadhyay, Vahid Dabbaghian

**Affiliations:** 1 The Modelling of Complex Social Systems (MoCSSy) Program, The IRMACS Centre, Simon Fraser University, Burnaby, Canada; 2 Department of Criminology, Simon Fraser University, Burnaby, Canada; 3 Department of Geography, Simon Fraser University, Burnaby, Canada; 4 Department of Mathematics, Simon Fraser University, Burnaby, Canada; 5 School of Computing Science, Simon Fraser University, Burnaby, Canada

**Keywords:** Homelessness, Complex social system, Fuzzy logic, Fuzzy Cognitive Map, Network analysis

## Abstract

**Background:**

The forces which affect homelessness are complex and often interactive in nature. Social forces such as addictions, family breakdown, and mental illness are compounded by structural forces such as lack of available low-cost housing, poor economic conditions, and insufficient mental health services. Together these factors impact levels of homelessness through their dynamic relations. Historic models, which are static in nature, have only been marginally successful in capturing these relationships.

**Methods:**

Fuzzy Logic (FL) and fuzzy cognitive maps (FCMs) are particularly suited to the modeling of complex social problems, such as homelessness, due to their inherent ability to model intricate, interactive systems often described in vague conceptual terms and then organize them into a specific, concrete form (i.e., the FCM) which can be readily understood by social scientists and others. Using FL we converted information, taken from recently published, peer reviewed articles, for a select group of factors related to homelessness and then calculated the strength of influence (weights) for pairs of factors. We then used these weighted relationships in a FCM to test the effects of increasing or decreasing individual or groups of factors. Results of these trials were explainable according to current empirical knowledge related to homelessness.

**Results:**

Prior graphic maps of homelessness have been of limited use due to the dynamic nature of the concepts related to homelessness. The FCM technique captures greater degrees of dynamism and complexity than static models, allowing relevant concepts to be manipulated and interacted. This, in turn, allows for a much more realistic picture of homelessness. Through network analysis of the FCM we determined that *Education* exerts the greatest force in the model and hence impacts the dynamism and complexity of a social problem such as homelessness.

**Conclusions:**

The FCM built to model the complex social system of homelessness reasonably represented reality for the sample scenarios created. This confirmed that the model worked and that a search of peer reviewed, academic literature is a reasonable foundation upon which to build the model. Further, it was determined that the direction and strengths of relationships between concepts included in this map are a reasonable approximation of their action in reality. However, dynamic models are not without their limitations and must be acknowledged as inherently exploratory.

## Background

### Homelessness

Homelessness is a complex social problem with a variety of underlying economic and social factors such as poverty, lack of affordable housing, uncertain physical and mental health, addictions, and community and family breakdown. These factors, in varying combinations, contribute to duration, frequency, and type of homelessness. To be fully homeless is to live without shelter; however, many experience partial homelessness that can include uncertain, temporary, or sub-standard shelter. Homelessness is difficult to define, thus governments struggle with uncertainty when creating and implementing policies they hope will effectively manage or eradicate this problem.

Levels of government, in countries like Canada, add to the complexity of dealing with homelessness. Being governed at three different levels, federal, provincial, and municipal, requires high levels of agreement to effectively create and administer policies. In Canada, each level of government is responsible for different facets of homelessness. The federal government, responsible for the whole of Canada, creates and administers policies and funding for aboriginal peoples (a segment of Canada’s population over-represented in homeless counts), seniors, and social housing, as well as transfers funds to the provinces to help pay for their social programs. The provincial government, responsible for needs of the provinces and territories, creates and administers policies regarding mental illness, addictions, welfare, minimum wage laws, landlord and tenant acts, and child protection services and shares responsibility with the federal government for seniors and social housing. The municipal governments, are seen as the hands or arms of the provincial government, and are technically not responsible for homelessness; however are often involved in choosing sites for social housing, supporting emergency shelters and hospital emergency wards, as well as providing support, in a variety of ways, to facilitate these initiatives. The fact that there is no comprehensive national housing strategy to co-ordinate these levels of government often leads to inadequate policies and funding that fall far short of meeting the country’s housing needs [1]. This lack of coordination towards policy and funding for homelessness has recently come to the attention of courts in Canada who have begun to make decisions which support shelter as an essential right for Canadians [2]. The UN Special Rapporteur on adequate housing in Canada has also strongly urged the federal government to commit sufficient funding to create a national housing strategy by working with the provinces and territories [3].

**Figure 1:**
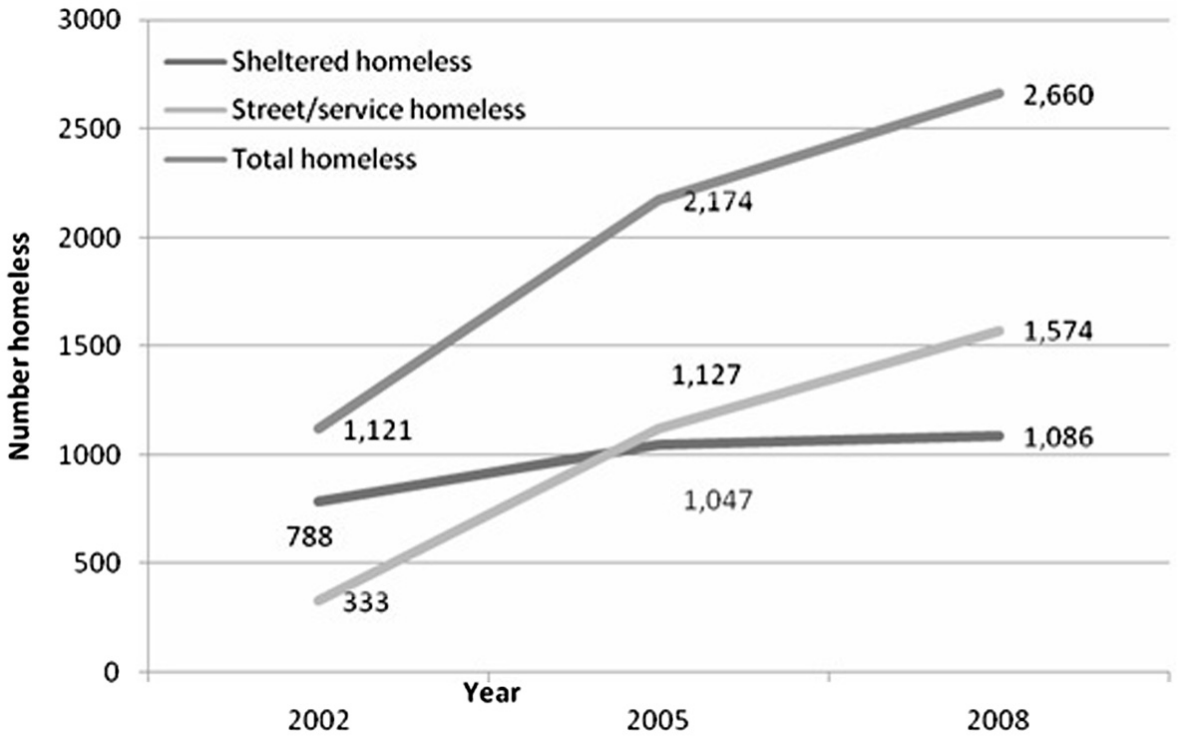
Homeless count in Metro Vancouver.

Metro Vancouver is one city in Canada which conducts a comprehensive homeless count every three years [4]. Counters make every effort to include in the count those considered sheltered homeless (individuals who spend nights in shelters, safe houses, transition houses, hospitals, jails, remand centres, and detox/recovery facilities) and those who are unsheltered homeless (individuals who spend their nights unsheltered on streets, in parks, or at drop-in programs). Counts are shown in Figure 1.

It becomes apparent that if the complex and oft-times chaotic experiences such as job loss that lead to family breakdown, mental illness, and drug/alcohol addiction, which may lead to homelessness, were better understood then social policies and procedures which constitute “best practices” would be more effective in reducing and preventing homelessness [5]. Fuzzy logic and fuzzy cognitive maps are especially useful for modelling complex social problems due to their inherent ability to capture and model vague concepts and values [6]. In relationship to homelessness, syllogisms such as, “if there is a lack of affordable housing, then there will be a significant increase in homelessness” can be accurately modelled by assigning a value to the parameter based on the retrieved linguistic terms taken from existing empirical literature. In this way greater meaning, which captures and aggregates the nuances of the stressors and protective factors, is given to the existing empirical literature related to homelessness. This also allows the complex social issue to be graphically described in a manner which may be more readily understood. This, in turn, may then help social policy-makers to refine their decision-making, leading to effective changes in social policies with the goal of reducing homelessness.

Fuzzy logic (FL) is a mutli-valued logic technique that is approximate. Rather than using traditional logic theory where binary sets have a two-valued logic (i.e., true, 1, and false, 0), fuzzy variables have a truth-value between 0 and 1, allowing them to be valued between absolutely true and absolutely false. Using linguistic variables, taken from empirical literature that describes the effect each factor in a knowledge system has on the others, FL can be used to convert the effects into values between 0 and 1. Once determined, these values can then be input into a graphical representation of the system containing all factors with directed lines (edges) showing the calculated strength of the causal relationship between them. This graphical representation is known as a fuzzy cognitive map (FCM). A brief description of the techniques, with an example is presented in the subsequent subsection.

### Fuzzy Cognitive Map (FCM)

**Figure 2:**
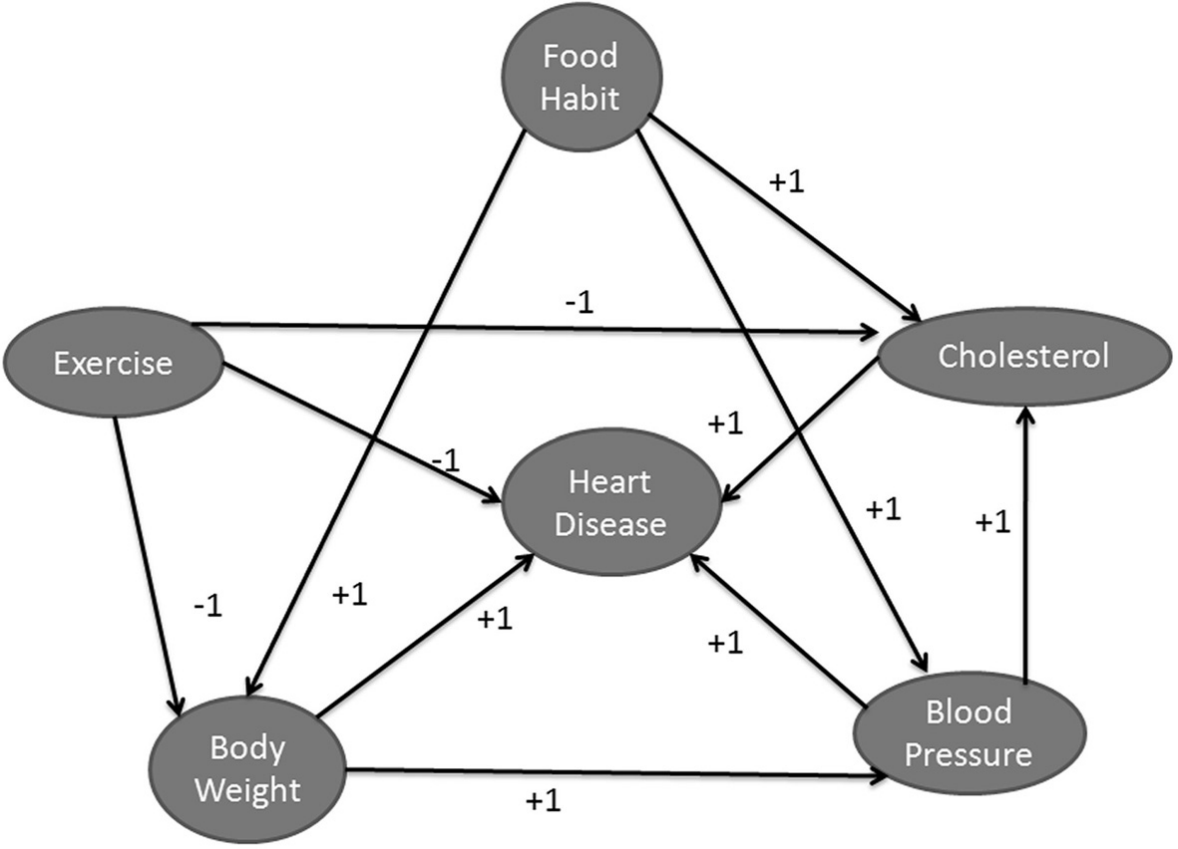
Example of a simple FCM to assess heart disease.

The FCM is a framework used for modelling interdependence between concepts in the real-world [7]. This is achieved by graphically representing the causal reasoning relationships between vague or un-crisp concepts [6,8]. FCMs allow scientists to construct virtual worlds in which some of the complex and interdependent concepts of a scenario can be captured and their interactions or causal relationships modelled. Knowledge representation in these maps has an acquisition-processing trade-off. FCMs, by providing a fuzzy graph structure for systematic causal propagation and ease in processing fuzzy knowledge, are applicable in soft-knowledge domains such as the social sciences. At the core of the FCM structure are the concepts to be studied and modelled. Concepts can be understood to represent actors or the parts of the environment which have impact on some phenomenon of interest (and each other), such as those included in the simple FCM of heart disease illustrated in Figure 2.

The concepts, determined empirically, which relate to heart disease in this model include: exercise (E), food habits (FH), cholesterol (C), blood pressure (BP), and body weight (BW). The links, directionally joining the concepts, represent the fuzzy causal relationships.

Concepts which have no impact on other concepts are not represented via links on the map, however are represented in the subsequent constructed adjacency matrix *W* and denoted, 0.

$$W=\begin{array}{ll} \begin{array}{l} E\\ \text{FH}\\ C\\ \text{BW}\\ \text{BP}\\ \text{HD} \end{array} & \overset{\begin{array}{cccccc} \;E & \;\text{FH} & C & \;\text{BW} & \text{BP} & \text{HD} \end{array}}{\left(\begin{array}{cccccc} 1 & 0 & -1 & -1 & 0 & -1\\ 0 & 1 & 1 & 1 & 1 & 0\\ 0 & 0 & 1 & 0 & 0 & 1\\ 0 & 0 & 0 & 1 & 1 & 1\\ 0 & 0 & 1 & 0 & 1 & 1\\ 0 & 0 & 0 & 0 & 0 & 0 \end{array}\right)} \end{array}$$

As can be seen in Figure 2, there is no direct effect of **BW** on **C** and therefore no link is drawn between these two concepts. The weight values {−1,0,1} are used at this stage for simplicity and testing the FCM and are later refined through the application of empirical linguistic terms and modifiers processed through FL.

The use of an FCM is particularly advantageous for graphically representing the interacting relationships of concepts which appear in phenomena related to social science, political science, organizational theory, military science, and international relations [8]. The connection matrix, *W*, may also be defined algebraically, demonstrating the influence concepts have on one another [7].

Let us denote the *i*^*th*^ concepts of a system as *C*_*i*_. Then the value *A*_*i*_, of a concept *C*_*i*_, expresses the quantity of its corresponding physical value. The FCM converges to a steady state when:

(1) $$|A_{i}(k+1)-A_{i}(k)|\leq \varepsilon$$

At each step, the value *A*_*i*_ of a concept is influenced by the values of concepts-nodes connected to it and is updated according to the following formula:

(2) $$A_{i}(k+1)=f\left(\overset{N}{\underset{j=1}{\sum }}\;A_{j}(k)W_{\text{ji}}\right),\text{with}\;W_{\text{ii}}=1,$$

where *A*_*i*_(*k*) is the value of concept *C*_*i*_ at step k, *A*_*j*_(*k*) is the value of concept *C*_*j*_ at step k, *W*_*ji*_ is the weight of the interconnection from concept *C*_*j*_ to concept *C*_*i*_ and *f* is the threshold function used to bound the transformation to a limit cycle. In this example, *f*(*x*) is a *sign* function defined in MATLAB [9] with the following functionality:

(3) $$f(x)=\left\{\begin{array}{ll} -1, & x<0\\ 0, & x=0\\ 1, & x>0 \end{array}\right)$$

Following our heart disease example, consider: the concept, E, is active for some individual. Therefore, *E*=1. No information is available for all other concepts in the map. Therefore, *FH*=0, *C*=0, *BW*=0, and *BP*=0. This is expressed by a vector *C*_1_ = (1,0,0,0,0,0). According to equations 2 and 3, the processing is listed in Table 1.

**Table 1:** FCM processing when excercise = 1

*C*_1_*W*	=	(0,0,−1,−1,0,−1)	→	(1,0,−1,−1,0,−1)	=	*C*_2_	
*C*_2_*W*	=	(0,0,−1,−1,−1,−3)	→	(1,0,−1,−1,−1,−1)	=	*C*_3_	
*C*_3_*W*	=	(0,0,−2,−1,−1,−4)	→	(1,0,−1,−1,−1,−1)	=	*C*_4_	⇔*C*_3_

The right arrow indicates the threshold function operation in Equation 3. The above results demonstrate that it takes four steps for the system to converge to a stable state (limit cycle). The vector *C*_4_ demonstrates that the increase in E eventually leads to decreases in C, BW, BP, and HD.

The FCM created for our study provides a graphical description of homelessness and facilitates increased understanding of this complex social problem. Through simulation, the usefulness of such a model is demonstrated and implications for its use in policy decision-making are explored. As shown, FCMs related to complex social problems, allow for refinement of knowledge through graphical understanding and simulations that may be useful in improving social policies with the goal of reducing homelessness.

## Methods

### Virtual common-sense map of homelessness

**Figure 3:**
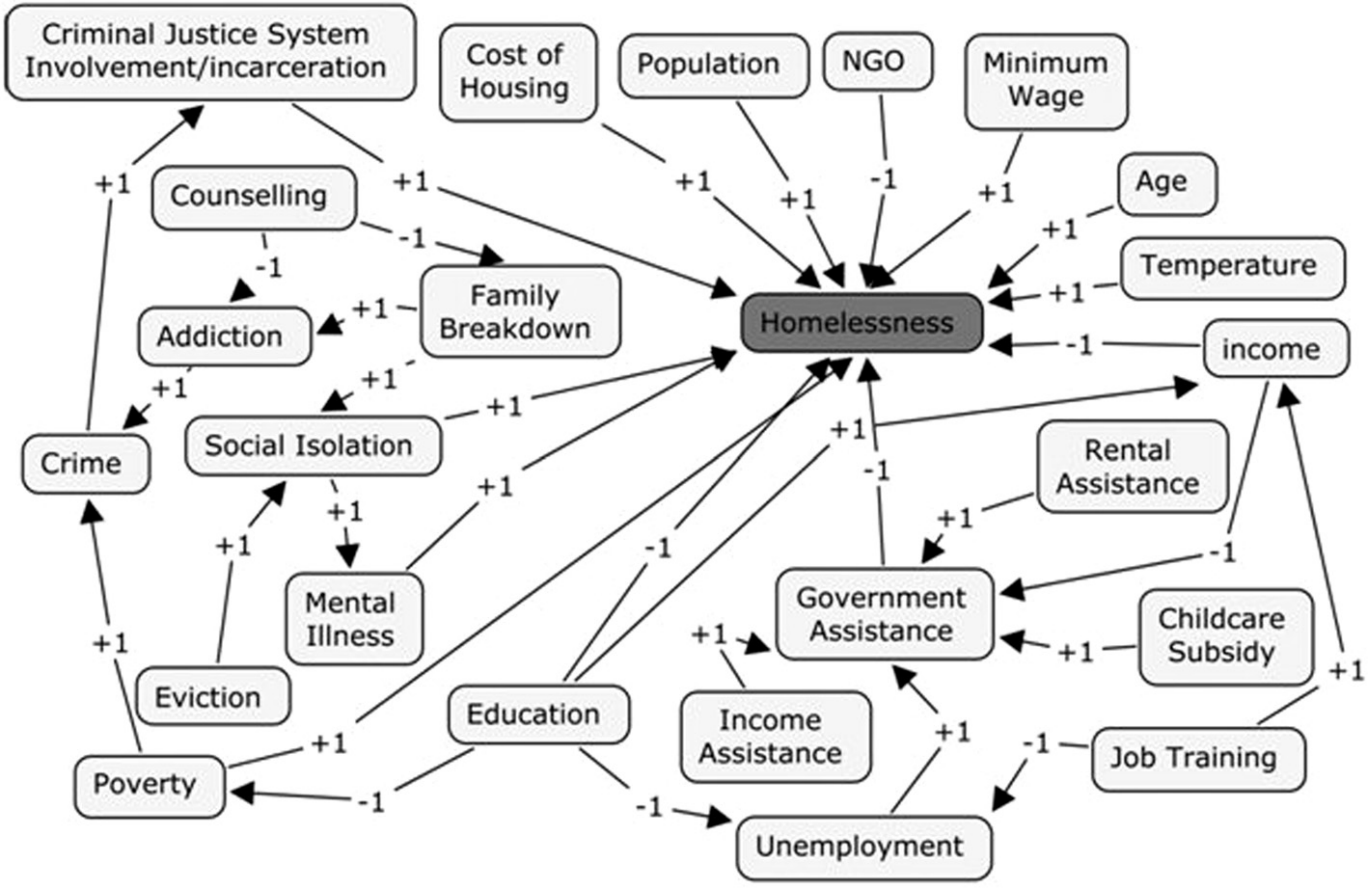
Virtual common-sense map of homelessness.

First a virtual common-sense map was built based on the researchers’ personal and historical knowledge of the factors which they perceived to affect homelessness. Using homelessness as the central hub of the map, concepts which directly or indirectly, positively or negatively affected homelessness, and each other, were linked through directed edges. Each edge was assigned a weight depending on whether the antecedent concept exerted a positive effect (+1) or a negative effect (−1) on the consequent concept (Figure 3). Three prototypical cases were then developed and the model was run to ensure it would function in accordance with the determined relationships prior to the actual weights on the edges being refined through a literature search for the linguistic terms.

#### Experimentation: Virtual common-sense map

Experimentation with the virtual common-sense model was conducted to ensure that it would perform as expected and reach a stable state after iterating prior to the input of the actual weight values. Sample cases were constructed with the goal of describing an extreme case, most likely to result in homelessness; an extreme case, least likely to result in homelessness; and a middle case, more closely representing the possibilities of the real world, in which the likelihood of homelessness would be uncertain, see Table 2. 

•*Case 1*: In this scenario, the protective factor of rental subsidy was incapable of preventing the negative social factors, criminal justice system involvement, addictions, and mental illness from overwhelming the model - resulting in certain homelessness.

•*Case 2*: In this scenario, the protective factors of education and increased income resulted in the elimination of the need for non-government assistance and a decrease in the likelihood of criminal justice system interaction. This is a highly likely outcome given that those with higher incomes and education are better able to identify and seek help for their mental illnesses which increases the likelihood that they will avoid incarceration. However, the strength of income and education as protective factors against increasing mental illness is shown to be ineffective and the level of mental illness continues to rise. Despite the increase in mental illness, education and income will ensure an ongoing ability to provide shelter, resulting in homelessness being an extremely unlikely outcome.

•*Case 3*: In this scenario, at the end of iteration 1, the effects of addiction, prior criminal justice system involvement, and family breakdown are held at bay by the protective factors of income, education and counselling. However, due to the known cumulative negative effects of addiction, social isolation increases, signalling the likelihood that, over time, there will be an increased possibility of family breakdown and greater challenges controlling the addiction resulting in the increased likelihood of crime. Iteration 2 demonstrates the actions of all the concepts present in iteration 1 continuing to exert force on the model with the addition of an increase in mental illness caused by the ongoing addiction resulting in an increasing likelihood of homelessness. As the model continues to iterate, the addictions contribute to increasing social isolation and criminal behavior resulting in a greater likelihood of family breakdown. At this point the protective factors of education, income and counselling are overwhelmed by the ongoing addictions and resulting mental illness and crime and the likelihood of homelessness rises. However, given that education and income continue to exert force, homelessness is not a certainty.

**Table 2:** Summary of expected outcome, concepts activated and iteration process for three sample cases

**Case**	**Concepts activated**	**Outcome**
Case 1: Extremely likely to result in homelessness	Criminal justice system involvement, addictions, mental illness, rental subsidy	Iteration 1: homelessness = 1; all other concepts, stable. *Conclusion*: extremely likely to result in homelessness.
Case 2: Extremely unlikely to result in homelessness	Education, mental illness, non-government organization, and income assumed to be high	Iteration 1: homelessness = -1; increase in education; decrease in poverty, unemployment, and government assistance; all other concepts, stable. Iteration 2: homelessness = -1; increase in education and mental illness; decrease in crime; and all other concepts, stable. Iteration 3: homelessness = -1; increase in education, and mental illness; decrease in crime and criminal justice system involvement; all other concepts stable. *Conclusion*: extremely unlikely to result in homelessness.
Case 3: Uncertain likelihood of homelessness	Criminal justice system involvement, addiction, family breakdown, increased income, education, and social systems network.	Iteration 1: homelessness = -1; increase in addiction, criminal justice system involvement, family breakdown, income, counseling, crime, and social isolation; decrease in poverty, unemployment, and government assistance. Iteration 2: homelessness = +1; increase in addiction, criminal justice system involvement, family breakdown, income, counseling, crime, and social isolation; decrease in poverty, unemployment, and government assistance; all other concepts, stable. *Conclusion*: uncertain likelihood of homelessness.

Given the fully explainable results of the model and the fact that it was able to achieve stability after iterating, it was determined that the model functioned properly, and the process of refining the concepts through the search of timely empirical literature was conducted.

### Fuzzy Cognitive Map of homelessness supported by empirical studies

**Figure 4:**
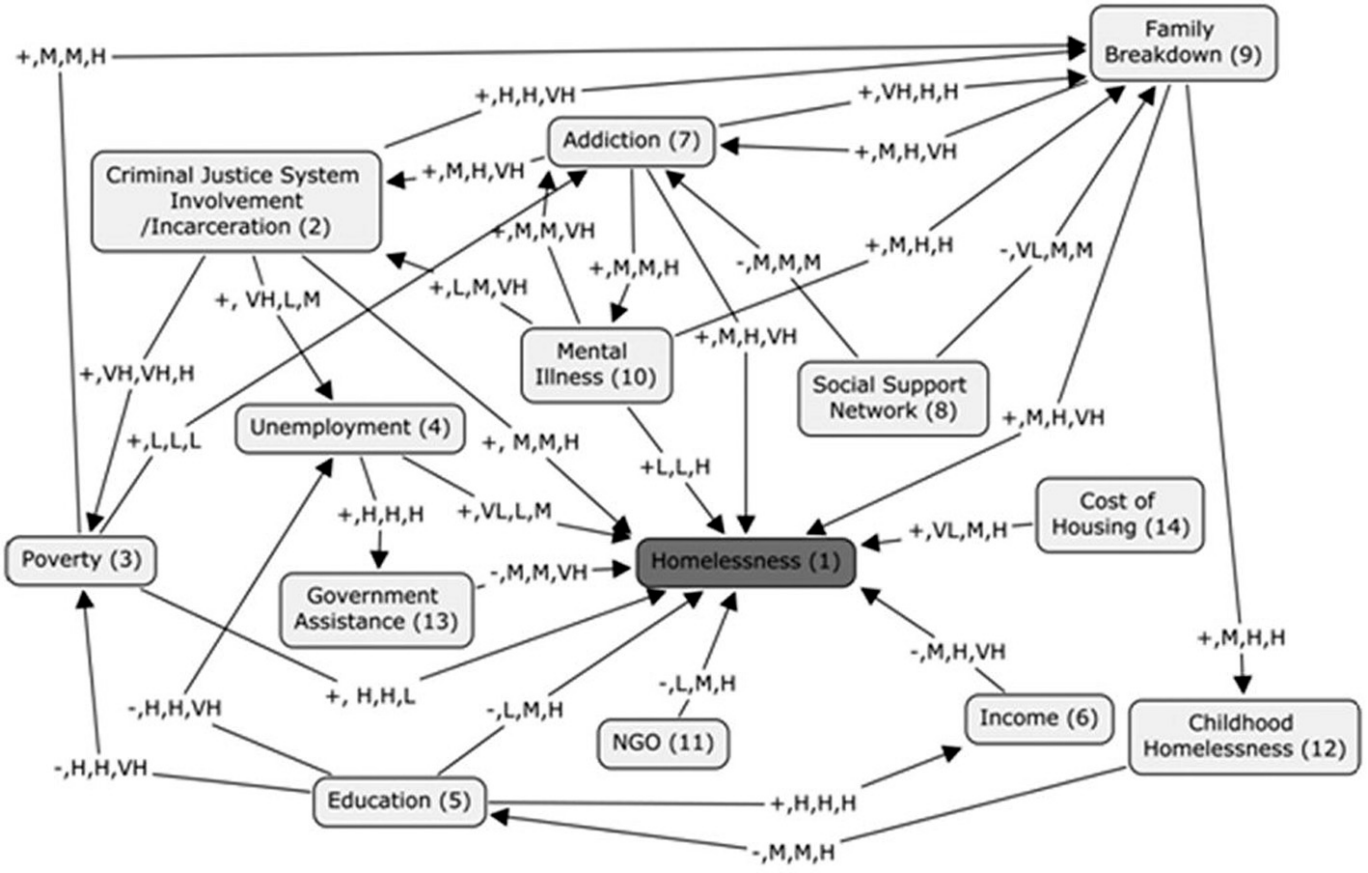
Fuzzy Cognitive Map with qualitative weighted edges.

To refine the edge weights on the FCM, timely, empirical literature was searched. The original causal map was referred to for the paired concepts such as, education and homelessness. These linked terms were then searched using the academic search engine, Google Scholar. Numerous articles were retrieved and scanned for each pair of linked concepts using only recently published (since the year 2000), peer reviewed, empirical articles. This culminated in the capture of three linguistic statements per concept pair for use in refining the map (see Table 3). Linguistic statements were required to be in the antecedent - consequent form as earlier described. In the process of searching, paired concepts were refined (edges and concepts added and removed from the virtual common-sense map Figure 3 after though deliberation with research team) resulting in a final map of 14 concepts and 31 edges (Figure 4). To maintain the semantic consistency amongst various concepts, Oxford Canadian Dictionary [10] was followed.

**Table 3:** Linguistic terms and the references

**Edge of the FCM**	**Type of Influence**	**Reference**	**Keywords**	**Linguistic terms**
CJS(2) → Homelessness(1)	Positive	[11]	Significantly Associated	High
		[12]	Appear to increase	Medium
		[13]	Much more likely	High
CJS(2) → Poverty(3)	Positive	[14]	significantly increase	Very high
		[15]	Most important	High
		[16]	Significantly positive	Very High
CJS(2) → Unemployment(4)	Positive	[17]	Significantly lower	Very high
		[18]	Can diminish but not necessarily	Low
		[19]	Relatively low	Medium
CJS(2) → Family Breakdown(9)	Positive	[18]	Significant correlates	High
		[20]	Tremendous strains	Very high
		[21]	Important causal factor	High
Poverty(3) → Homelessness(1)	Positive	[5]	Significantly and independently	High
		[22]	Run a great risk	High
		[23]	May experience	Low
Poverty(3) → Addiction(7)	Positive	[24]	Good chance	Low
		[25]	Might be more limited	Low
		[26]	Likely to	Low
Unemployment(4) → Homelessness(1)	Positive	[11]	More likely	Medium
		[27]	Primary risk factor	Low
		[28]	Did not predict	Very low
Unemployment(4) → Government Assistance(13)	Positive	[29]	Play an important role	High
		[30]	Substantial	High
		[31]	Substantially	High
Education(5) → Homelessness(1)	Negative	[5]	Thereby increase	Medium
		[32]	Rectricts	Low
		[22]	Run a great risk	High
Education(5) → Poverty(3)	Negative	[33]	Strong positive correlation	High
		[34]	Vital	Very high
		[35]	Powerful instrument	High
Education(5) → Unemployment(4)	Negative	[36]	Significantly increases	Very high
		[37]	Much higher	High
		[38]	Strong determinant	High
Education(5) → Income(6)	Positive	[39]	Strongly correlated	High
		[40]	Strong positive	High
		[41]	Thwarted/an important means	High
Income(6) → Homelessness(1)	Negative	[13]	Significantly and strongly	Very high
		[42]	Strong	High
		[41]	Most effective	Medium
Addiction(7) → Homelessness(1)	Positive	[5]	Independently associated	Medium
		[43]	Key factor	High
		[44]	Statistically significant	Very high
Addiction(7) → CJS(2)	Positive	[11]	Major contributor	High
		[45]	More likely	Medium
		[46]	Extensive	Very high
Addiction(7) → Mental Illness(10)	Positive	[47]	Increased risk	Medium
		[48]	Strong evidence	High
		[49]	More likely	Medium
Addiction(7) → Family Breakdown(9)	Positive	[50]	Critical	Very high
		[51]	Significant connection	High
		[51]	Strong connection	High
Social Systems Network(8) → Addiction(7)	Negative	[52]	Less likely	Medium
		[53]	Benefit	Medium
		[54]	Lower levels	Medium
Social Systems Network(8) → Family Breakdown(9)	Negative	[55]	Small, short lived	Very low
		[56]	Effective	Medium
		[57]	Effective	Medium
Family Breakdown(9) → Homelessness(1)	Positive	[58]	More prominently	Very high
		[59]	Significant proportion	High
		[60]	Increased risk	Medium
Family Breakdown(9) → Addiction(7)	Positive	[50]	Profound effect	Very high
		[50]	Usually	Medium
		[51]	Strong connection	High
Family Breakdown(9) → Childhood Homelessness(12)	Positive	[61]	Highly predictive	High
		[62]	Important role	High
		[63]	Most common	Medium
Mental Illness(10) → Homelessness(1)	Positive	[5]	Significantly and independently	High
		[44]	Not significant	Low
		[45]	A risk factor	Low
Mental Illness(10) → CJS(2)	Positive	[64]	Significantly more	Very high
		[65]	May trigger	Low
		[45]	Independently associated	Medium
Mental Illness(10) → Addiction(7)	Positive	[66]	Significantly more likely	Very high
		[67]	Remain problematic	Medium
		[49]	Common and of concern	Medium
Mental Illness(10) → Family Breakdown(9)	Positive	[68]	Significant correlates	High
		[69]	Increases chances of	Medium
		[70]	Strongly associated	High
Non-Government Assistance(11) → Homelessness(1)	Negative	[71]	Crucial	High
		[72]	Address needs	Low
		[73]	Considerable	Medium
Childhood Homelessness(12) → Education(5)	Negative	[74]	At risk	Medium
		[75]	Restricts	Medium
		[76]	Strong evidence	High
Government Assistance(13) → Homelessness(1)	Negative	[77]	Widely used to support	Medium
		[78]	Associated	Medium
		[79]	Most effective	Very high
Cost of Housing(14) → Homelessness(1)	Positive	[80]	Positive/negative and significant	High
		[44]	Not important	Very low
		[81]	Implicated	Medium
Poverty(3) → Family Breakdown(9)	Positive	[82]	Not uncommon	Medium
		[83]	Associated with	Medium
		[84]	The bulk of responsibility	High

To calculate the quantitative weight values for each edge, first the qualitative weight values for each of the retrieved linguistic terms was assessed. A Likert-type scale was devised to determine the qualitative weight of each linguistic term. The values, Very Low (VL), Low (L), Medium (M), High (H), and Very High (VH) were used to categorize each term. We only consider five qualitative values for the sake of simplicity. However, the scale could be less or more than five, depending on the intricacies of the system under consideration. Consensus on meaning was achieved through discussion and vote. This process resulted in a scale of ordered and ranked values for each concept pair. For example, it might be stated in one peer-reviewed study that the effect of concept A on concept B was, “profound”; whereas another article may state that the effect was, “significant”. These statements, “profound” and “significant”, would be then ranked on the Likert- type scale in reference to their absolute meaning as well as their relative meaning. Thus, “profound”, would be valued as VH and “significant” would be valued as H. In the case of disagreement or uncertainty regarding the precise meaning of the words, Oxford Dictionary Online was referenced for definitions and synonyms. A word bank was constructed during this process listing all the retrieved terms for both comparative reference and to ensure consistency in the rankings, see Table 4. Once the different qualitative weight values were determined for each linguistic term, they were then collected into their groups of three and applied to the revised FCM.

**Table 4:** Categorization of linguistic terms extracted from literature

**Very low**	**Low**	**Medium**	**High**	**Very high**
Did not predict, not important, small and short lived	Might be more limited, likely to, a risk factor, address needs, good chance, can diminish but not necessarily, lack, leads to, disadvantage, to restriction, leads to, likely, may experience, may trigger, might be, not significant, primary risk factor, risk, risk factor	Appear to increase, associated, at risk, benefit, common and of concern, considerable ongoing, cope, effective, implicated, increase risk, increases chances of, independently associated, less likely, lower levels, more difficult, more likely, most common, most effective, not uncommon, relatively, relatively low rates, remain problematic, restricts, thereby increase, usually, widely used to, support, associated with	Crucial, highly predictive, important role, important causal factor, important means, key factor, major contributor, most important, much more likely, much higher, play important role, positive and significant, powerful, powerful instrument, significant correlates, significant, independent significantly associated, significant proportion, statistically significant, strong, strongly associated, strong connection, strong correlation, strong determinant, strong effect, strong evidence, substantial, bulk of, positive/negative and significant, significant connection, strong positive, strong positive correlation, run a great risk, thwarted	Critical evidence indicates, extensive, more prominently, most effective, profound, significant and positive, significantly and strongly, significantly increase, significantly lower, significantly more, significantly more likely, statistically significant, tremendous, very high, vital

**Figure 5:**
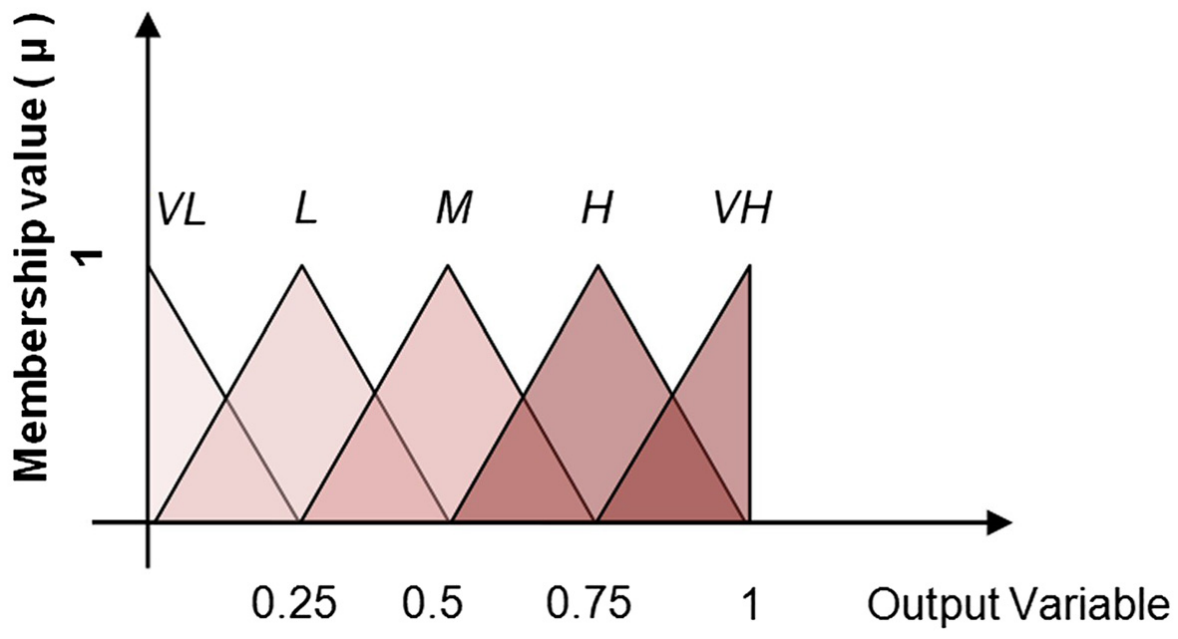
Triangular membership function.

Subsequent to the information from the literature review having been transferred to the FCM, the resulting map contained the concepts, the antecedent - consequent relationships indicated via edges, the weight value of each edge (five qualitative, linguistic terms - VL, L, M, H, VH), and the sign value showing the type of the influence (+ or −). Following the application of the qualitative values to the FCM the values were then converted to quantitative weight values using FL theory. Each link was first expressed as a fuzzy rule then used in the Fuzzy Inference System (FIS) to generate a crisp numeric value. For example, if the linguistic term retrieved from the literature was: “The impact of concept A is profound on concept B”. It would then be converted to: “The impact of concept A is VH on concept B”. This graded statement would then be transformed using the rule statement: 

The linguistic term ON is a binary variable. VH is defined using the triangular fuzzy membership function, as shown in Figure 5. ON denotes the presence of the concept and VH denotes the weight value (qualitatively). For simplicity sake, triangular membership functions have been used as suggested in [85]. Interested readers can find more detailed explanation on membership functions in [86]. 

**Figure 6:**
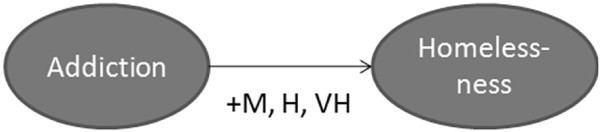
Impact of addiction on homelessness.

•*Example 1:* As explained in the previous section, all qualitative values assigned to the edges came from the literature review. As shown in Figure 6, “addiction” has a positive impact on homelessness. This means that an increase in addiction in a society will lead to an increase in levels of homelessness. The three linguistic terms related to “addiction”, extracted from the literature, were converted to the fuzzy notion of rules as follows: 

•The degree of impact was then converted from its qualitative value (M, H, VH) to its quantitative value of 0.648 using FL concepts as described in [87]. All three studies indicated that as levels of addiction increase they exert a positive effect resulting in increases in levels of homelessness. Therefore, it can be stated that addictions affect homelessness by a factor of +0.648.

**Figure 7:**
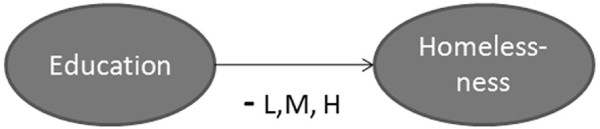
Impact of education on homelessness.

•*Example 2*: As shown in Figure 7, education has a negative effect on homelessness. This means that with higher levels of education in a society there will be lower levels of homelessness. Therefore, the impact of education on homelessness is modeled as negative - increases in education lead to decreases in homelessness. All literature scanned indicates that as education rises, homelessness falls. The first study stated that the impact of education on homelessness was *low*, the second, *medium*, and the third, *high*. This information is captured to construct a rule base for a Fuzzy Inference System (FIS). For each edge, we constructed an individual FIS and the defuzzified value, in this case 0.5, is assigned to the edge. More information about the procedure can be found in [87-90]. 

**Figure 8:**
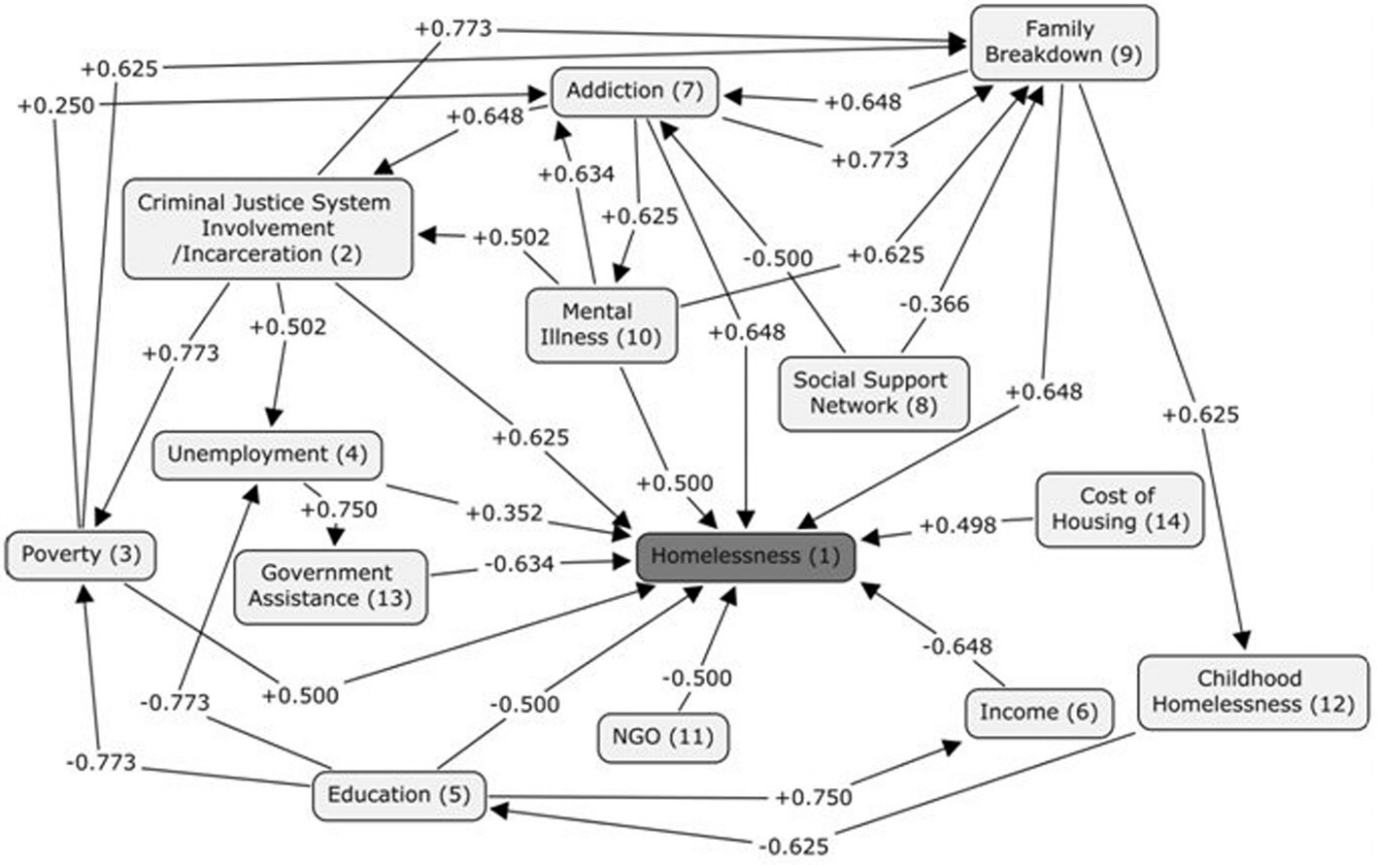
Fuzzy cognitive map with calculated quantitative weights assigned to edges.

•Similarly, each edge was given a quantitative weight by converting the qualitative values gleaned from the literature search. Once all links on the map had been fully articulated with the rankings of each of the 93 linguistic terms (three for each link), we refined the virtual FCM (shown in Figure 4) by substituting quantitative values for the previous qualitative values (see Figure 8).

## Results

### Experimentation with the weighted Fuzzy Cognitive Map

Experimentation with the weighted FCM was conducted, (see Algorithm 1), to ensure that it would perform as expected and that the map had captured the dynamics of the factors which affect levels of homelessness. We applied $\text{tanh}=\frac{e^{2x}-1}{e^{2x}+1}$ as the transformation function *f* of Equation 2. This choice is made as we are interested in understanding the impact of increase or decrease of initial concept values on the overall stability of the map [91]. 

Prototypical scenarios, similar to those used for the simplified FCM (Figure 3), were constructed with the goal of finding the extreme case most likely to result in homelessness, the extreme case least likely to result in homelessness and several middle cases, more closely representing the possibilities present in the real world, where levels of homelessness are less certain.

The output of each prototypical case was interpreted through knowledge gleaned during the literature search/scan and the opinion of the criminologist-researcher on the team. Each example case had a variety of concepts activated at varying levels. The models were then permitted to iterate as necessary to reach a stable state (no further movement, positive or negative, for all concepts in the model). Final iterations are reported for each model. 

•*Case 1: Most likely to result in homelessness.* The concepts of addiction, family breakdown, government assistance, and mental illness were activated at levels considered sufficiently high to dominate the system leading to certain homelessness as shown in Table 5. It has been empirically determined that these concepts are often found together and often precede homelessness [52,70,83]. Addiction and mental illness are often co-morbid and both commonly precede family breakdown [51]. During times of increased addiction and mental illness in society it is the usual reaction of the government to put into place policies and funding which will address these problems [93].

**Figure 9:**
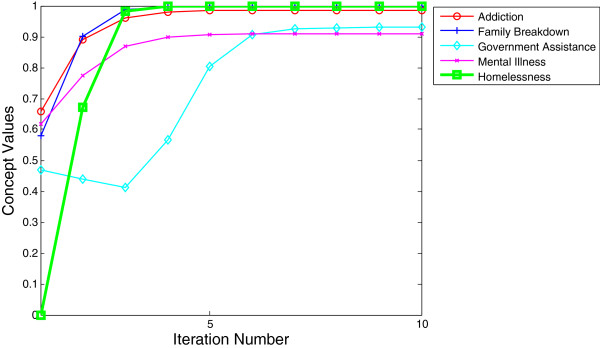
Activated concepts at levels most likely to result in homelessness with graphical representation of impact of concepts on levels of homelessness over time.

•Tracking the effect of these concepts at strengths set to approximately 0.50, the graph initially shows that government assistance is at a lower rate and then sharply rises to address the increasing levels of addiction, mental illness, and family breakdown in the modeled society. However, it takes little time before the triple threat of addiction, mental illness and family breakdown overwhelm the system and levels of homelessness rise dramatically where they remain at a steady, high rate (indicated by the flat line at the top of the graph, Figure 9).

•*Case 2: Least likely to result in homelessness.* The concepts of addiction, education, income, family breakdown, and social network support were activated at levels considered sufficiently high to dominate the system leading to a certain outcome of no homelessness as shown in Table 6. In this case, the protective factors of education, income, and social network support protect society from the negative effects of addiction and prevent homelessness. The link between higher levels of education and higher levels of income have been well documented [72]. Given that education prepares individuals to think creatively and to problem-solve, it is surmised that those with higher levels of education would have a greater ability to negotiate the complex rules that often are associated with government assistance. Those who are wealthy and educated are also much more likely to be capable of identifying and acquiring the services they might need, such as being able to pay for family counseling rather than being wait-listed for government supplied family counseling.

**Figure 10:**
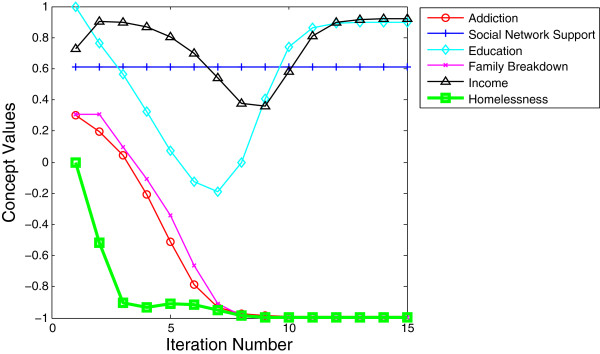
Activated concepts at levels least likely to result in homelessness with graphical representation of impact of concepts on levels of homelessness over time.

•From Figure 10, it is noted that this model shows a initial dip in levels of income and education in the first iterations as society attempts to deal with the addictions and threat to family cohesion that result from the addictions. However, very quickly, the protective factors of income, education, and social network support overwhelm the negative factors and the threat of homelessness diminishes and remains at levels close to zero (as indicated by the flat line at the bottom of Figure 10). Over time, the threat of family breakdown is also eliminated and income and education both rise back to their initial levels.

•This second model demonstrates the critical importance of factors such as income - which lead to health, acquisition of knowledge, better food and health care; and education - which lead to wealth and all the positive factors which wealth can purchase. Though addictions are shown as present in this modeled society, the low levels are unable to overwhelm the model. Through model testing it became apparent that levels of addiction lower than 0.30 often fail to overwhelm the positive factors, as long as social support and education are both present at fairly high levels, see Figure 10. Much of the empirical literature support this [41,59,78]. Those with high levels of social support such as family, church, social groups, community groups, school friends and community friends are often better able to weather threats such as addictions and family breakdown.

•*Case 3: Uncertain outcome of homelessness.* In this model, we activated low levels of addiction and social network, high levels of education and income, and moderate levels of family breakdown as shown in Table 7. In this case, the protective factors of education and income delay the onset of homelessness but are insufficiently strong to prevent rising levels as the model iterates. Over time, due to family breakdown and the diminishing social network support, addictions begin to rise and as addictions rise, the likelihood of homelessness rapidly increases. This model demonstrates, once again, the importance of family and social support as well as the incredibly negative effects of drug addiction, both as a cause and result of family breakdown.

**Figure 11:**
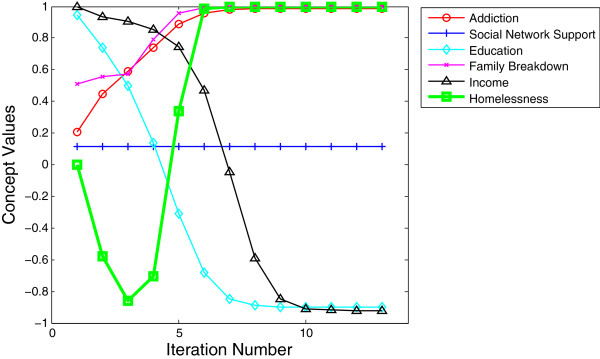
Activated concepts at levels most closely representing a typical real-world case with graphical representation of the impact of concepts on levels of homelessness over time.

•As in the case of the common-sense map of homelessness (Figure 3), this final model (Figure 11), acted in a manner which was fully explainable based on information acquired during the literature search and prior knowledge of the research team. This allowed for confidence that the model was functioning as it ought to and that we had captured not only a number of the integral aspects which contribute to homelessness, but that they were functioning in the direction and strengths which approximated real-life conditions.

**Table 5:** Simulating the result for case 1

	**Activated concepts**			
	**Addiction**	**Family breakdown**	**Government assistance**	**Mental illness**
Inital values	0.65	0.57	0.46	0.61

**Table 6:** Simulating the result for case 2

	**Activated concepts**				
	**Addiction**	**Social network support**	**Education**	**Family breakdown**	**Income**
Initial values	0.30	0.61	1.0	0.30	0.72

**Table 7:** Simulating the result for case 3

	**Activated concepts**				
	**Addiction**	**Social network support**	**Education**	**Family breakdown**	**Income**
Inital values	0.20	0.11	0.94	0.51	1.0

### Analysis of network concepts

The purpose of this network analysis is to compare the degree of impact each of the concepts exerts on the model. During network analysis, we varied the initial value of a single concept from 0.1 to 1 while keeping the initial values of all other concepts at a static level; except for the concept representing homelessness. After several iterations, the value of homelessness was recorded. Then, for each factor, a plot of the value of homelessness versus the initial value of the concept was recorded. Ideally, for a factor with a positive effect on homelessness, the value of homelessness should increase as the value of the factor increases, gradually converging to a positive value. Concepts which have the reverse - a negative effect on homelessness, should demonstrate a decrease in homelessness as they are increased. Concepts which have higher convergent rates should demonstrate a greater impact on levels of homelessness.

**Figure 12:**
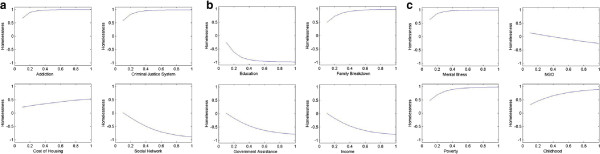
Comparison of the affects of individual concepts on levels of homelessness (a) shows the impact of Addiction, Criminal Justice System, Cost of housing and Social Network on Homelessness (b) highlights the impact of Education, Family Breakdown, Government Assistance and Income on Homelessness and (c) depicts the impact of Mental Illness, NGO, Poverty and Childhood hardships.

To conduct the network analysis we first set the initial values for all concepts at a level of 0.5 and checked the levels of homelessness after 5 iterations. At this level and number of iterations, the majority of the plots resulted in a straight line at a value of +1. This told us that the initial value of the factor (0.1 to 1) made no difference on levels of homelessness and, obviously, was no help to our analysis. After analyzing the map, we tried reducing the level of the initial values for all concepts as well as reducing the number of iterations. Through a gradual reduction process we found that by setting the initial concept values at 0.01 and running three iterations we were able to generate reasonable and useful plots (see Figure 12) which could then be compared for effects on levels of homelessness.

Plots can be examined in pairs or groupings so that the effect of the concepts on levels of homelessness can be compared for both intensity and speed. For example, in comparing the plots for, “Addictions”, and, “Cost of Housing”, it can be seen that they both are monotonically increasing. However, the plot for “Addictions” demonstrates a more dramatic increase, resulting in a quicker convergence to +1 than does the plot for “Cost of Housing”. Therefore it can be concluded that addictions have a greater impact on homelessness than does cost of housing.

**Figure 13:**
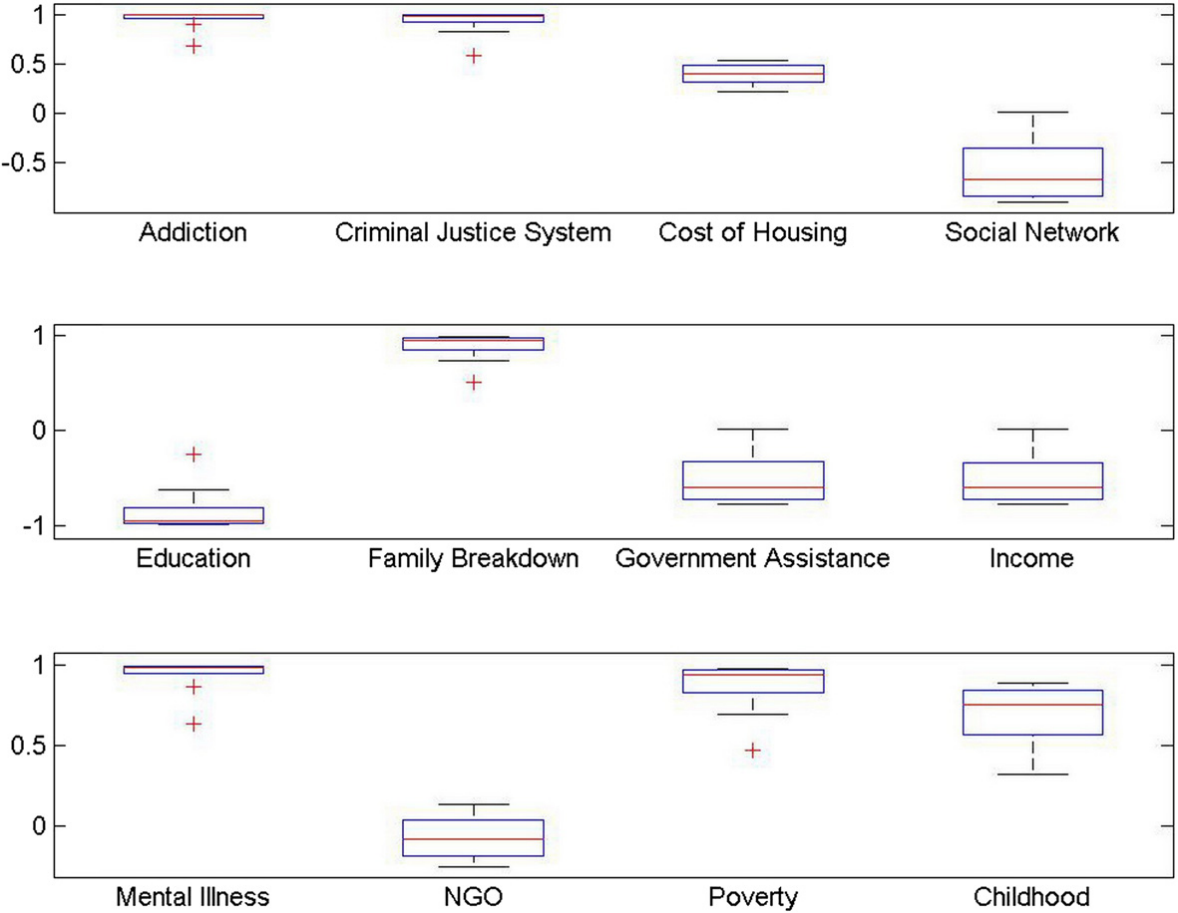
Boxplot comparison of the affects of individual concepts on levels of homelessness.

Another way to visually analyze the impact of various factors on homelessness is through *box plota* (see Figure 13). Making the same comparison, “Addictions” to “Cost of Housing”, it can be seen that the plot of “Addictions” has a narrower median and longer lower quantile. The size of the box determines the variability of concepts, for instance, the size of the box of “Cost of Housing” is greater than size of the box of “Addictions” indicating that the impact of housing cost is more variable and hence not a strong indicator [94].

#### Measure of centrality

Another approach to analyze the most influential factor is through *measures of centrality*. There are also other measurements for analyzing an FCM, but here we focus on this property. In this subsection, we describe the results of the analysis based on two types of centrality: degree centrality and closeness centrality. Degree centrality of each node/concept, in a given weighted and directed graph, is defined as the sum of the absolute values of the weights of the outgoing and incoming edges [8,95]. For the node, *x*, of the graph *G*=<*V*,*E*> the degree centrality is mathematically defined as:

(4) $$\underset{\forall y\in V}{\sum }|w_{\text{xy}}|+|w_{\text{yx}}|$$

where *w*_*xy*_ and *w*_*yx*_ are the weights of the edge from *x* to *y* and the edge from *y* to *x*, respectively. Degree centrality of a graph indicates how strongly a concept node in a FCM affects other concept nodes of the graph [96].

Closeness centrality of a node is the inverse of the sum of the lengths of the shortest paths between that node and all other nodes. For the node, *x*, of the graph *G*=<*V*,*E*>, the closeness centrality is mathematically defined as:

(5) $$\frac{1}{\underset{\forall y\in V}{\sum }|d_{\text{xy}}|}$$

where *d*_*xy*_ denotes the length of the shortest path from node *x* to node *y*. Closeness centrality indicates how quickly a concept node affects other nodes of the FCM [96].

*Note*: For closeness centrality the distance measured between each pair of nodes is the inverse of the weight of the corresponding edge in the FCM. If there is no edge between nodes then the distance from the one node to the second node would obviously be infinite. Since the FCM is not strongly connected, the length of the shortest path for some pairs of nodes is, in fact, infinite. This then causes the closeness centrality for that node to drop to zero. For example, the length of shortest path for each node to the node, “Cost of Housing”, is infinite. This makes the centrality of all nodes to be zero. To conquer this problem, we choose a numerical value which is large enough to be considered as an infinite value. Since the distance measure between each pair of nodes is defined as the inverse of the weight between the nodes of the FCM, the greatest distance between each two nodes would be 4. This value is corresponding to the edge between “poverty” and “addiction”, whose weight is 0.25. The FCM has 14 concepts, thus each path of the FCM will, at most, have 13 edges. Therefore, the length of each path will be at most 4×13=52, which is still an overestimation of the paths in the graph. Regarding this value, we picked 100 as an large enough value. This approach is similar to the Big-M method described in operation research theories [97]. Please note that changing 100 to a greater value, may change nodes’ closeness centrality, but the order of the nodes’ closeness centrality will not change.

The result of the degree and closeness centrality computation in our FCM is displayed in Table 8. As shown, the concept “Education” has the greatest degree centrality while the concept “Cost of Housing” has the least. This means that “Education” gives and receives the greatest direct influence on all other concepts, whereas “Cost of Housing” gives and receives the least. Closeness centrality was determined to act similarly to degree centrality in that “Education” has the greatest amount of degree centrality whereas “Cost of Housing” has the least. This means that “Education” exerts the greatest force on the map in reference to closeness centrality with changes in “Education” resulting in the most prominent changes in the other concepts. Likewise, changes in “Cost of Housing” would result in the least amount of change in all other concepts. These results are consistent with the results of the overall experiment.

**Table 8:** Degree centrality and closeness centrality of every concept

**Concepts**	**Degree**	**Closeness**
	**centrality**	**centrality**
Criminal Justice System Involvement	3.0485	9.9514
Poverty	2.0451	8.3195
Unemployment	2.3763	9.0566
Education	5.4201	11.1514
Income	1.3978	8.3441
Addiction	3.7027	9.9533
Social Support Network	0.8656	9.1302
Family Breakdown	2.2862	9.9533
Mental Illness	2.2609	9.9446
NGO	0.5000	8.3472
Childhood Homelessness	1.2500	8.3445
Government Assistance	1.3844	8.3443
Cost of Housing	0.4984	8.3194

## Discussion

This study demonstrates the efficacy of using FCM to graphically represent and simulate the actions and interactions present in the social, personal, and structural factors related to homelessness. The FCM is particularly suited to modeling this type of problem due to its ability to incorporate vast amounts of information, synthesizing what is known about a problem and then allowing for meaningful simulations. The FCM is particularly suitable due to its dynamic nature and ability to simulate potential policy changes and show predicted outcomes on levels of homelessness. Further, the FCM helps to identify those factors that exert the greatest impact in a complex system, in this case: affordable/appropriate housing, access to social support services for those with addictions/mental illness, family support for those with children, positive community support and rental supplements.

The problem of homelessness is really situated in factors that occur at the micro-, meso-, and macro-levels of society; future research should aim to refine the FCM by sorting factors into their appropriate levels thereby allowing differentiation between what the individual is potentially capable of controlling and that which he or she is not. This would allow for clearer identification of where government policy changes would have the greatest effect. Future refinements must also capture the effect of time. Many factors affect the system differently as time progresses (i.e., unemployment insurance) and this would help to make the system more closely replicate reality. Future maps may also wish to include factors which affect the system but which did not make it into this one such as early brain injury in childhood, sexual/physical/emotional abuse in childhood, and learning disabilities - all of which have been shown to affect levels of homelessness.

The initial construction of this map demonstrated the disparity between the empirical truth of homelessness and what the researchers had learned over a lifetime of media and social propaganda. This has implications for government policy-making and, again, demonstrates the usefulness of FCMs for describing complex social problems such as homelessness.

## Conclusion

The FCM built to model the complex social system of homelessness reasonably represented reality for the sample scenarios. This provided evidence that FCMs are a viable alternative for conceptualizing homelessness and that a literature search of peer reviewed, academic literature is a reasonable foundation upon which to build the model. Further, it was determined that the direction and strength of relationship between concepts included in this map are a reasonable approximation of their action in reality. However, the concept, *homelessness*, in this study, is used as a consequent variable. In reality, many of the concepts including *homelessness* concept could be an antecedent concept resulting in more complex loops. The flexibility of limiting the complexity is one of the advantages of constructing and using FCMs for social science research.

Dynamic modeling does, however, have it’s limitations and this work should be regarded as purely exploratory. For one, by basing our concepts off of peer reviewed literature that was searched semi-systematically there is a possibility of not capturing all possible terms. Future work should search for papers and terms in a similar fashion as systematic or scoping reviews where inclusion and exclusion criteria are highly scrutinized and analyzed by several research team members. A second limitation concerns the interpretation of the results from the FCM. FCMs, and dynamic models more broadly, have the luxury of experimenting with problems in an environment that is encapsulated from the real-world. It should be noted that every societal issue carries with it its own contextual element that cannot always be captured by a modeling environment. Further, FCMs do not fully replicate the mirco-level interactions that may prove to be powerful in determining meso- and macro-level outcomes. Future work should aim to incorporate these influences in to their models and interpretations as best possible. Lastly, dynamic models are exploratory and we can not reasonably assume that outcomes presented in this research will be realized in the real world.

This research provides empirical support for the usefulness of this model, not only for researchers and social scientists, but for others who reside within a society where homelessness is experienced. This model is based on a limited collection of published, peer reviewed scholarly articles but despite this limitation, does justify the use of FCM techniques as a useful tool to analyze the complex situation of homelessness. The role of FCM for the purpose of modelling complex social systems has been strongly supported by this research and should continue to be utilized in future studies.

## Competing interests

The authors declare that they have no competing interests.

## Authors’ contributions

VKM and VD conceived the idea and formulated mathematical model. TW, SN, PG, RC and VKM implemented the computational model. HKM, CF wrote the paper along with VKM. All authors critically analyzed the simulations, reviewed the manuscript, read and approved the final version.

## Pre-publication history

The pre-publication history for this paper can be accessed here:

http://www.biomedcentral.com/1472-6947/13/94/prepub
